# How can we reduce the rate of Violent-Sexual Crimes? An attempt at street design

**DOI:** 10.1371/journal.pone.0341372

**Published:** 2026-01-21

**Authors:** Yiyao Zhang, Zikai Jin, Shu Zhuang, Shengjie Zhao, Li Ling

**Affiliations:** 1 School of Civil Engineering and Architecture, College of Science & Technology Ningbo University, Ningbo, Zhejiang, China; 2 Nanjing University of the Arts, Nanjing, Jiangsu, China; Universidade Estadual de Maringa, BRAZIL

## Abstract

Well-designed streets can foster a harmonious social environment. Street crime rates are closely related to street design, and Violent-Sexual Crime (VSC) is a significant public order concern. While most existing studies address the impact of single environmental factors on street crime, a comprehensive assessment that integrates multiple factors is lacking. As an exploratory study, this research aims to propose and preliminarily validate a multi-factor street design framework aimed at mitigating VSC. First, the Fuzzy Delphi Method (FDM) was used to construct street design guidelines that affect the Violent-Sexual Crime rate, with scoring criteria for street design factors formulated based on the FDM results. Subsequently, Google Street View (GSV) and Semantic Segmentation were utilized to capture the streetscape of the study area. To empirically test this framework, the study conducted an in-depth analysis of fifteen streets, randomly selected from a focused area in London using QGIS. The results indicated a negative correlation between higher environmental scores and lower VSC rates within the studied setting, providing initial, context-specific evidence for the potential utility of the proposed design guidelines. Although findings are primarily applicable to similar urban environments, the methodology offers a transferable model for future research in broader contexts.

## Introduction

Streets ar vital urban elements for traffic, open space, and diverse activities. Well-designed streets enhance residents’ quality of life and urban vitality by attracting people [1], fostering healthy interactions [2] and well-being [3], and improving perceived security [4]. Safe, walkable streets with good pedestrian conditions promote healthier lifestyles and sustainable development [5]. Specific design elements (e.g., benches, greenery, maintenance) directly impact environmental quality and safety [6–8].

However, street crime significantly degrades the environment and quality. High crime rates induce fear, harming individual psychology and community cohesion [9]. Physical disorder signs (e.g., broken windows, graffiti, vacant lots) indicate social control breakdown [10], damage street quality, cause panic [11], and can foster further crime [12]. Therefore, effective street design is crucial to mitigate these issues.

There have been a lot of discussions in the theoretical field on the prevention of crime by street space patterns. Jane Jacobs first proposed the theory of intervening in crime through urban design, in which she argued that street safety depended on the existence of the “Street Eye” [13]. Based on this, Oscar Newman proposed the Defensible Space Theory, which argued that spaces with a hierarchy of domains, with natural surveillance capabilities, and with a sense of belonging for their inhabitants can reduce crime [14]. Meanwhile, C. Ray Jeffery created Crime Prevention Through Environmental Design (CPTED), which aimed to reduce crime through the use of “Access control”, “Territorial reinforcement”, “Surveillance”, “Maintenance” and “Activity support” [15]. Rob Krier noted that the problems in the streets can be attributed to “homes backing onto the street, poorly arranged garages, isolated neighborhood recreation areas, and the lack of street trees and vegetation” [16]. As evidenced by the aforementioned theories, scholars have extensively examined streets in terms of their physical composition and scale, with the aim of exploring design interventions for street environments and spaces and reducing crime rates.

Existing research often examined the impact of single environmental factors on crime. Studies showed correlations between lower crime rates and higher street tree canopy cover, better streetscape greenery [17], increased street light density [18] and more surveillance cameras [19]. Elements like bus stops, graffiti and decorations also associate with crime occurrence [20–22].

While research often focused on robbery [19] or burglary [23], Violent-Sexual Crimes (VSC) were highly prevalent (e.g., second highest rate in London) [24] and severely impacted victims, causing profound psychological harm and fear [25,26]. VSC often involves violence alongside sexual offenses [27]. This study defines VSC as encompassing acts from minor assault and harassment to injury, homicide, rape, sexual assault, and related violent acts during sexual offenses.

In addition, there have been a few studies on the correlation between the environment and VSC, usually focusing on the effects of objective natural environmental factors such as time, space [28] and temperature [29]. Street safety has a significant association with VSC. Mohamed et al. examined the relationship between sexual harassment and street network layout and found higher rates of sexual harassment in places with complex street layouts and high pedestrian traffic [30]; Koskela et al. explored the relationship between street space, safety, and gender, noting that women were concerned about whether the space was monitored [31]. Further, in studies addressing street environments and Violent-Sexual Crimes, most focused on the impact of a single design element. For example, focusing on the “bus stop”, Hoor compared sexual harassment on public transportation in two cities and proposed design guidelines to help law enforcement agencies take strict action against perpetrators to mitigate the harms of sexual harassment on public transportation [32]. Hewitt et al. used Crime Pattern Theory and Social Disintegration Theory to study the characteristics of sexual crime locations and found that sexual crimes occur more often in bars and school areas [33]. However, such studies lacked an examination of the relationship between overall street environment design and crime rates.

In summary, existing evidence indicates that street environment design can influence crime occurrence. Moreover, existing studies have only examined the relationship between single environmental factors and crime rates. This study analyzes the correlation between each environmental factor of the street and VSC by considering street environmental factors in an integrated approach, thus proposing a street environmental design method that can help reduce the rate of VSC.

## Methods

### Street design factors affecting Violent-Sexual Crimes

Based on the correlation factors between the street environment and crime compiled by He et al. [34], combined with expert interviews, the study summarizes the descriptions and theoretical foundations of street environment factors influencing VSC in Table 1.

**Table 1:** Street environment factors and theories related to Violent-Sexual Crimes.

Dimensions	Factors	Theoretical/empirical foundations	Sources
Territorial reinforcement	Bus stops	Streets with bus stops have higher rates of sexual assault because bus stops not only bring in crowds but are also good places for offenders to escape. Most sexual harassment occurs in buses and around stations. While stations bring more surveillance, they also provide opportunities for sexual offenders to commit crimes in crowded situations.	Melo et al. [35] Orozco-Fontalvo et al. [36]
Surveillance	Shrubs	Well-greened streets with a wide variety of greenery can reduce crime rates.	Lin et al. [17]
	Trees	Well-maintained street greenery has a significant effect on reducing violent crime.	
	Street lights	“Almost half of sexual assaults occur at night and two-thirds of sexual assaults occur in the early morning hours”, and molestation crime rates increase if the environment is dark.	Cohn,1993 [37]
	Motor vehicles/non-motor vehicles	Areas where motor vehicles are crowded or parked indiscriminately may reduce the power of natural surveillance across the street.	Doeksen [38]
	Surveillance cameras	Sexual assaults often occur in poorly monitored locations.	Ceccato [28]
Maintenance	Potholes in the street	Physical signs of environmental degradation can induce potential deviance and crime.	George L. Kelling and James Q. Wilson [10]
	Litter;	A decaying physical environment (e.g., trash, graffiti, abandoned buildings) indicates that the physical environment of the community is neglected, and the community often becomes a high crime area.	Cohen, 2014 [11]
		Dilapidated exterior/ Cracked brick or concrete	
	Personalization of the property; House or yard decorations; Garden	Neighborhoods with more personalized decorations for houses or yards have lower crime rates. Offenders may not choose streets with strong management to commit crimes.	Perkins et al. [22]
Activities	Shops	The higher the density of commercial facilities, the higher the probability of assault.	Smith and Clarke.[39]
	Clubs/Bars	Entertainment establishments with alcohol have higher rates of sex crimes.	Johnson et al. [40]
	Schools	School proximity may indicate a high-sex crime area. More crime occurs in places easily accessible to the public, and the flow of people creates opportunities for crime, typically in shopping centers, high schools, transportation hubs, etc.	Hewitt et al. [33] Zeng et al. [41]

### Applying the Fuzzy Delphi method to construct street design guidelines to reduce the rate of Violent-Sexual Crimes

Fuzzy Delphi Method (FDM) enhances the traditional Delphi technique by integrating fuzzy set theory to address expert opinion ambiguity and improve consensus convergence [42,43]. Experts provide anonymous three-point estimates (conservative, moderate, optimistic) to form Triangular Fuzzy Numbers (TFNs). Consensus is determined by calculating the geometric mean (G_i_) of these values and identifying the intersection of TFNs.

The purpose of using FDM was to obtain expert professional opinions on street design factors impacting VSC, ranked by their perceived degree of correlation, to construct locally consistent street design guidelines that are scientifically derived from the expert consensus obtained from FDM. According to Table 1, the following 13 street environment design factors were extracted to reduce VSC.

**Fig 1:**
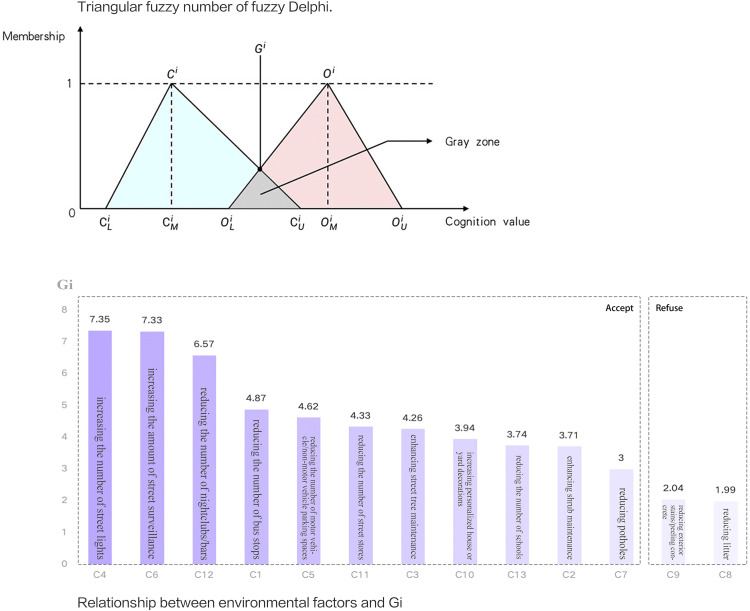
Triangular fuzzy number of fuzzy Delphi and the relationship between environmental factors and G_i._

In this phase, 10 experts who were long-term residents of London participated in the FDM process [44,45]. The panel was composed of ten specialists whose expertise collectively spans the key disciplines relevant to crime prevention through environmental design. The group included professionals with specialized knowledge in urban planning and public space design (3), environmental criminology and CPTED principles (2), environmental psychology and human behavior (2), building technology and environmental engineering (2), and project management and community engagement (1). This multidisciplinary composition ensured a comprehensive evaluation that integrated perspectives on spatial design, criminal opportunity, human perception, technical implementation, and stakeholder coordination. FDM questionnaire questions presented in S1 Questionnaire. The panel’s expertise remained appropriate for establishing CPTED-based design guidelines, as the core methodology prioritizes spatial interventions. Future iterations could benefit from multidisciplinary collaboration to integrate complementary perspectives on VSC-specific risk factors. In this study, the opinions of experts were collected through questionnaires and TFNs were created as follows:


$$\text{TFN}=({\text{C}}^{\text{i}},\;{\text{G}}^{\text{i}},\;{\text{O}}^{\text{i}})$$ (1)

$${\text{C}}^{\text{i}}=\text{min}\left({\text{X}}_{\text{ij}}\right)$$ (2)

$${\text{G}}^{\text{i}}=\sqrt[{\text{n}}]{\prod_{\text{j}=\text{1}}^{\text{n}}\text{Xij}}$$ (3)

$${\text{O}}^{\text{i}}=\text{max}\left({\text{X}}_{\text{ij}}\right)$$ (4)

where i is the number of options; j is the number of experts; C_i_ is the bottom level of all experts’ evaluation values for option i; O_i_ is the upper limit of all experts’ evaluation values for option i; G_i_ is the geometric mean of all experts’ evaluation values for option i; X_ij_ is the evaluation value of j experts for option i. Outliers exceeding twice the standard deviation were removed. Factors failing the consensus threshold (60% of G_i_, set at 2.68) were eliminated. Fig 1 illustrates the fuzzy number of fuzzy Delphi and factor-G_i_ relationship, confirming validated criteria for guideline development.

### Process

**Fig 2:**
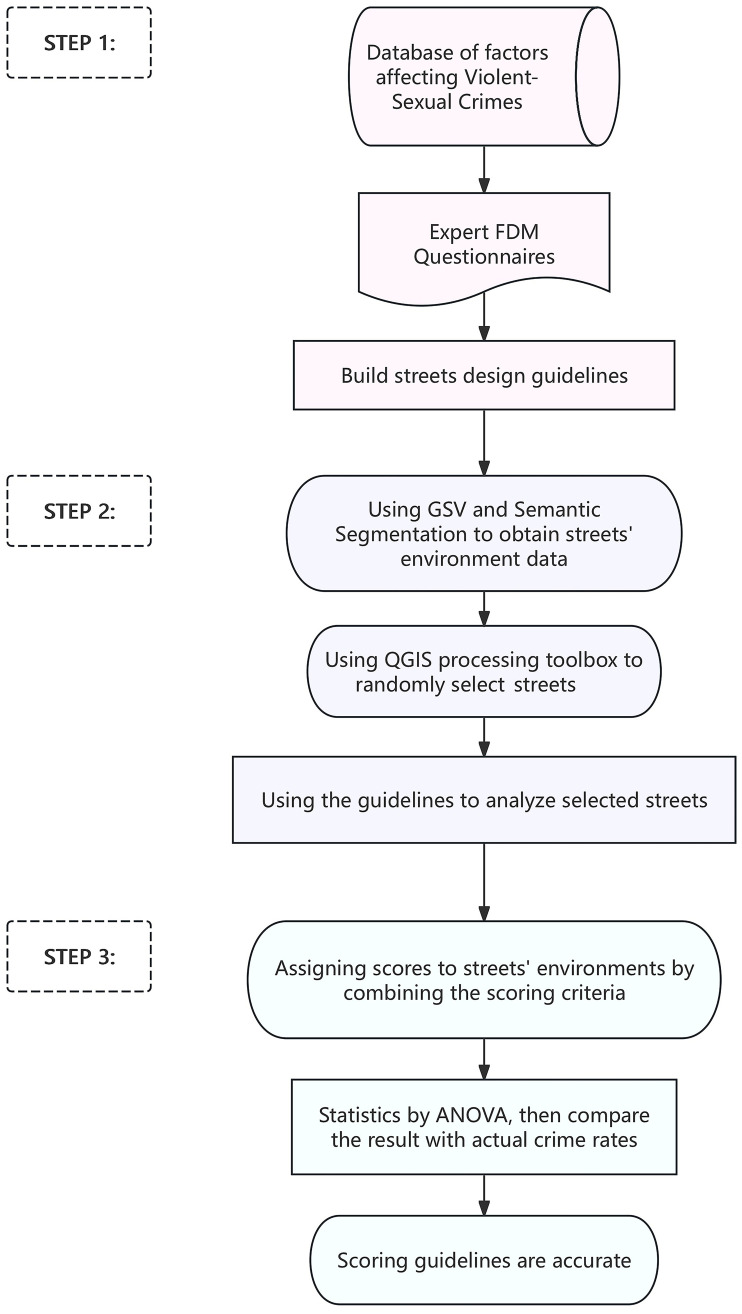
Validation experiment flow.

Examples were used to verify whether the street design guidelines were applicable to determine the Violent-Sexual Crime levels of different local streets. The process was as shown in Fig 2. Step 1, establish the scoring criteria for the factors affecting the environment of VSC. Step 2, collect data on VSC in residential areas of London, a representative city in the UK. Street images will be collected using Google Street View and Semantic Segmentation, and streets will be randomly selected using QGIS tools, ensuring that they are located in the same area and have similar regional environments. Step 3: Assign scores to the fifteen street environments using the scoring criteria. The scores are then compared with actual crime rates via ANOVA to validate the criteria.

### Street environment factor scoring criteria

Based on the environmental factor scale proposed by Abdullah et al. [46] and Hedayati et al. [47], combined with the FDM results, the scoring criteria of the street design factors affecting the VSC rate were established as shown in Table 2, and the scale was used to distinguish between the positive and negative degree of each factor in different streets.

**Table 2:** Scoring criteria of the street design factors affecting the Violent-Sexual Crimes rate.

Factors (scores 1–5)	Indicators
Bus stops	1 = Many bus stops (>3) 2=With 3 bus stop 3=With 2 bus stop 4=With 1 bus stop 5 = No bus stop
Shrubs	1 = No shrubs or trees 2 = Landscape elements eliminate over 80% of viewing opportunities 3 = Landscape elements eliminate over 60% of viewing opportunities 4 = Landscape elements eliminate over 40% of viewing opportunities 5 = Landscape elements eliminate over 20% of viewing opportunities
Trees	
Street lights	1 = No illumination in the area 2 = Lighting facility exists just along streets and pedestrian routes (20% lighting) 3 = Lighting facility exists along streets and main entrances (40% lighting) 4 = Lighting facility exists along streets; main entrances and yards (60% lighting) 5 = Lighting facility exists along streets and main entrances and near the building and attached to the building (80% lighting)
Motor vehicles/non-motor vehicles spaces	1 = Large number, disorderly parking 2 = Messy parking 3 = Low quantity, neatly parked 4 = Fewer in number, neatly parked 5 = No motor vehicle/non-motorized parking
Surveillance cameras	1 = No surveillance 2=With 1 surveillance camera 3=With 2 surveillance cameras 4=With 3 surveillance cameras 5 = Multiple surveillance cameras (>3), each area is in the monitoring range
Potholes in the street	1 = Many potholes in the road (>6) 2=With 5–6 potholes 3=With 3–4 potholes 4=With 1–2 potholes 5 = No potholes in the road surface
Personalization on the property; House or yard decorations; Garden	1 = Houses do not have personalized decorations and have poorly maintained yards 2 = Houses have 1 personalized decorations and generally well-maintained yards 3 = Houses have 2–3 personalized decorations and generally well-maintained yards 4 = Houses have 3–4 personalized decorations and generally well-maintained yards 5 = Houses have plenty of personalized decorations, and their yards are well maintained
Stores	1 = Large number of stores (>3) 2=With 3 stores 3=With 2 stores 4=With 1 store 5 = No stores
Clubs/Bars	1 = High number of nightclubs/bars (>3) 2=With 3 nightclubs/bars 3=With 2 nightclubs/bars 4=With 1 nightclub/bar 5 = No nightclubs/bars
School	1 = Large number of schools (≥2) 2 = Low number of schools (1) 3 = No school

### Source of crimes and environmental data

Reported crime data (Jan 2021-May 2025) were sourced from Metropolitan Police [24]. This study involved the retrospective analysis of existing, fully anonymized, and publicly available crime data. The crime data were sourced from the Police.uk database. As per the UK Government’s official stance and the design of this service, all data published on Police.uk is anonymized and does not contain any personal information about victims, witnesses, or offenders. The specific analysis dataset derived for this study is available in the Figshare repository (10.6084/m9.figshare.30539945). Street-level environmental factors were assessed using Google Street View (GSV), a validated data collection tool [34,48]. The study focused on North Lambeth, London, selected for its high VSC rates [49] and because its characteristics (e.g., relative ethnic homogeneity, economic stability) [50,51] helped control for major socio-demographic confounders, enabling a clearer examination of street-level environmental effects.

In addition, in the North Lambeth area, under the condition that the macro socioeconomic factors are roughly the same, then fifteen streets were randomly selected, and the streets within each area cover the street environmental factors that this study focuses on. Among them, an area is a spatial unit with clear geographical boundaries and socioeconomic homogeneity, and a street is the basic component of urban space, carrying social activities and environmental elements. Through such a selection, the variables are effectively controlled, which is helpful for in-depth analysis of the relationship between street environmental factors and VSC.

**Fig 3:**
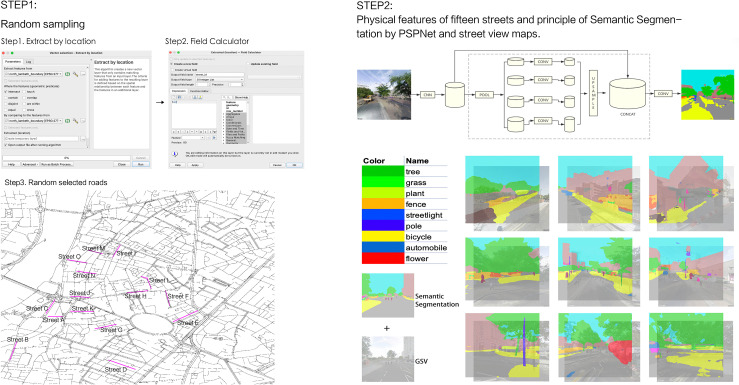
Study area and environmental data. Note: The base map was created by the authors using QGIS software (v3.40) with open-source boundary data from the Open Geography Portal of the Office for National Statistics. This map is an original creation for this study and is available under the CC BY 4.0 license.

The selection of fifteen streets within North Lambeth was conducted using a random sampling approach implemented in QGIS (v3.40) [52]. QGIS is an open-source software that has been used by many researchers to analyze geographic spaces [53,54]. All pedestrian-accessible street segments within the borough boundary were first extracted. To ensure comparability, segments outside a typical local street length range (approximately 100–300 meters) were excluded, focusing the analysis on a consistent scale of urban fabric. From this filtered pool, fifteen streets were then randomly selected using QGIS’s random selection utilities. This method aimed to provide a representative snapshot of street-level environments within the study area while mitigating selection bias and controlling for gross variations in street length. Similar sample sizes and street-level random sampling are established in exploratory built-environment research where detailed streetscape assessment is prioritized. The selected sample size is consistent with the scale of analysis employed in focused, street-level exploratory studies [55,56]. While this sample size provides a detailed, contextual understanding of environmental relationships, it may limit the statistical power to detect small effects. Similar small-scale, in-depth audits of street segments have been used as a valid methodological approach for preliminary investigations of fine-grained environmental relationships, where the primary goal is hypothesis generation and framework validation rather than broad generalization. At 5,542 points (every 50m), GSV panoramic images were captured in four cardinal directions (n = 19,297). Semantic segmentation, performed using a PSPNet model trained on ADE20K, classified pixels into 150 urban element categories (e.g., trees, grass, bicycles) for quantitative environmental analysis. The results of Semantic segmentation are presented in S1 Table. GSV images and segmentation maps were combined to mitigate detail loss and inform street scoring. The selected streets (Fig 3) captured diverse environmental conditions, from degraded to well-maintained.

According to the scoring criteria of the street design factors affecting VSC rate (Table 2), each factor in the study street was scored (shown in S2 Table).

### Violent-Sexual Crimes quantities on streets

The VSC rate for each street was calculated using the raw count of crimes occurring on that street during the 53-month study period. These raw counts were used as the primary metric in subsequent correlation and ANOVA analyses. This decision was based on data availability constraints and the exploratory nature of this study, which prioritizes the initial validation of a multi-factor environmental scoring framework. The crimes that occurred in each street during the 53 months are shown in S3 Table. The total number of VSC across the fifteen streets, in descending order, ranged from Street A (highest) to Street E (lowest).

## Results

### Comparison of crime rates by the streets

Office for National Statistics census data showed Lambeth’s total population is 317,600 with a resident density of 85 persons/8000 m² [57]. Given the area’s relatively uniform social, economic, cultural environment and population density, the number of street crimes is used as a proxy for the crime rate in this comparative analysis. S2 Table yields the VSC rate comparison: Street A (0.0008%)> Street B (0.0007%)>Street O (0.00058)> Street C (0.00056%)> Street N (0.00039%)> Street G (0.00026%)> Street L (0.000248%)> Street D (0.000245%)> Street M (0.000192%)> Street J (0.000189%)> Street F = Street K (0.000129%)> Street I (7.556E-5%)> Street H (4.723E-5%)> Street E (4.0932E-5%).

### Comparison of the correlation between environmental factors and crime rates by the streets

${\text{G}}_{\text{i}}$ in Fig 1 represents the degree of association of individual street design factors with crime rates, with higher weight values indicating a higher association of the factor with the crime rate. The study combined the weight (${\text{G}}_{\text{i}}$) from the FDM with the street-specific score (${\text{a}}_{\text{i}}$) for each factor (see S2 Table) to calculate an influence indicator (${\text{S}}_{\text{i}}$), which represents the contribution of a single design factor to the street crime rate. The formula was created as follows:


$${\text{S}}_{\text{i}}={\text{a}}_{\text{i}}*{\text{G}}_{\text{i}}$$ (5)

Then, the sigma notation was used to find the sum of the combined scores of all factors for a street:


$$\;\sum {\mathrm{\backslash nolimits}}_{\text{i}=\text{1}}^{\text{n}}{\text{S}}_{\text{i}}={\text{S}}_{\text{1}}+{\text{S}}_{\text{2}}+{\text{S}}_{\text{3}}+\ldots +{\text{S}}_{\text{n}}$$ (6)

where i = 1 and n = 11 (the number of factors retained). The data of each of the fifteen streets were calculated according to this formula, and the final scores ($\sum S_{i}$) are shown in Table 3. A higher $\sum S_{i}$ value indicates a greater presence of crime-deterring environmental factors on a street.

**Table 3:** Violent-Sexual Crimes rate (R) and the $\sum {\text{S}}_{\text{i}}\;$ of each street.

Order	Item	R (%)	$\sum {\text{S}}_{\text{i}}$
**1**	Street A	0.0008%	121.25
**2**	Street B	0.0007%	148.64
**3**	Street O	0.00058%	142.27
**4**	Street C	0.00056%	161.65
**5**	Street N	0.00039%	142.27
**6**	Street G	0.00026%	183.8
**7**	Street L	0.000248%	166.39
**8**	Street D	0.000245%	184
**9**	Street M	0.000192%	162.23
**10**	Street J	0.000189%	136.37
**11**	Street F	0.000129%	181.34
**12**	Street K	0.000129%	173.99
**13**	Street I	7.556E-5%	180.76
**14**	Street H	4.723E-5%	190.42
**15**	Street E	4.0932E-5%	212.58

### Analysis of ANOVA

ANOVA (F-test) determines significant differences between multiple groups using probability distributions. The p-value measures the probability of observing test results assuming the null hypothesis (H₀: no group difference) is true. A small p-value (<0.05) provides evidence for the alternative hypothesis (H₁: difference exists). ANOVA was used to compare the relationship between the number of VSC per month and $S_{i}$ for each street. Prior to conducting the ANOVA, descriptive statistics and tests for homogeneity of variances were performed. The descriptive statistics (see Table 4) revealed notable differences in the mean monthly counts of VSC across the fifteen streets. For instance, Street A had the highest mean (M = 4.82), while Street E had the lowest (M = 0.25). The test for homogeneity of variances (see S4 Table) indicated a significant Levene’s statistic (Levene’s statistic based on mean = 15.60, p < 0.001), suggesting that the assumption of homogeneity of variances was violated.

**Table 4:** Descriptive statistics for ANOVA.

Street	N	Mean Crime Count	Std. Deviation
**Street A**	53	4.8113	2.63889
**Street B**	53	4.2453	2.22664
**Street C**	53	3.3585	1.72197
**Street D**	53	1.4717	1.15365
**Street E**	53	0.2453	0.51537
**Street F**	53	0.7736	0.97352
**Street G**	53	1.5472	1.40830
**Street H**	53	0.2830	0.63177
**Street I**	53	0.4528	0.95204
**Street J**	53	1.1321	1.56939
**Street K**	53	0.7736	0.91234
**Street L**	53	1.4906	1.26526
**Street M**	53	1.1509	1.33584
**Street N**	53	2.3585	1.84072
**Street O**	53	3.4906	1.94761
**Total**	795	1.8390	2.07218

Consequently, Welch’s ANOVA was employed for further analysis (see S4 Table). The results were significant (Welch’s F (14, 295.50) = 45.76, p < 0.001), indicating statistically significant differences in crime rates among the streets. The standard ANOVA results also supported this conclusion (F (14, 780) = 49.92, p < 0.001; see Table 5). For example, Street A showed significant differences with Streets D, E, G, H, I, J, K, L, M, and N, but not with Street O. The complete post-hoc analysis results are presented in S4 Table. These findings demonstrate that the differences in crime counts across the studied streets are statistically significant.

**Table 5:** ANOVA for the number of cases per month on fifteen streets.

Source of Variance	SS	df	MS	F	P-value
Between Group	1611.127	14	115.081	49.916	<0.001^***^
Within Group	1798.264	780	2.305		
Total	3409.391	794			

Note: ^***^p < 0.001.

Based on the Games-Howell post-hoc test results from S4 Table, an analysis of the differences in mean monthly crime counts among the streets (A-O) was conducted. The post-hoc comparisons revealed that Street E differed significantly (p < 0.05) from all streets except Street H (Mean Difference = −0.038, p = 1.000) and Street F (Mean Difference = −0.528, p = 0.051). The differences were particularly pronounced (p < 0.001) when compared to the “high crime count” group (e.g., Streets A, B, C, O). Street D (M = 1.47, SD = 1.15) also exhibited widespread significant differences, showing statistically significant distinctions from most other streets, except when compared to Street F (Mean Difference = 0.698, p = 0.068), Street G (Mean Difference = −0.075, p = 1.000), and Street L (Mean Difference = −0.019, p = 1.000). Notably, no significant differences were found among Streets A, B, and C themselves (A-B: p = 0.997, A-C: p = 0.072, B-C: p = 0.598), forming a homogeneous group with higher crime counts. On the other hand, Street O, one of the streets with the highest means, differed significantly from the vast majority of streets (p < 0.001), with the exception of Street N (Mean Difference = 1.132, p = 0.144). Furthermore, Street H (M = 0.28, SD = 0.63), similar to Street E, showed significant differences from most streets in the “high crime” group, but not from streets with medium crime counts like F, I, and K. Street J (M = 1.13, SD = 1.57) occupied an intermediate position, showing significant differences only with some streets (e.g., E, H, O). These results clearly delineate a three-tier structure in the distribution of crimes among the streets: Streets E and H belong to a low crime count group; Streets A, B, C, and O form a high crime count group; and Streets D, F, G, I, J, K, L, M, and N constitute an intermediate transition group. This finding not only validates the significance of the overall ANOVA but also reveals the specific distribution patterns of crime counts across different streets.

## Discussion

**Fig 4:**
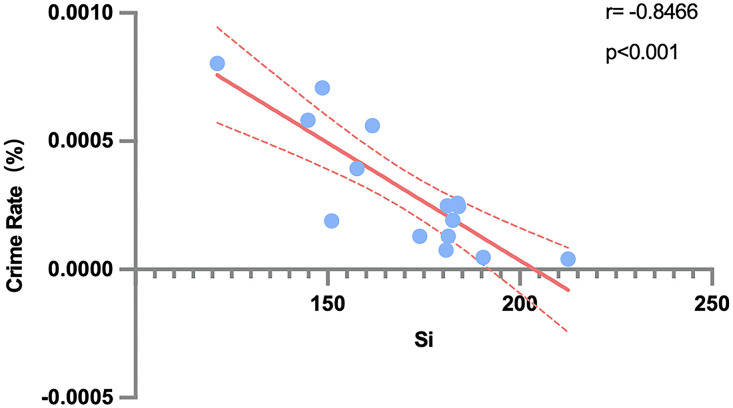
Pearson analysis of relationship between R and$S_{i}$.

This study proposes and provides a preliminary validation for a set of scoring criteria for street design factors aimed at mitigating Violent-Sexual Crime (VSC) rates. Each of the fifteen randomly selected streets was evaluated against these criteria (Table 2), generating a comprehensive environmental profile for each site (see S2 Table). To quantify the relationship between the overall street design quality and crime rates, a Pearson correlation analysis was performed between the aggregated street design score ($\sum {\text{S}}_{\text{i}}$) and the VSC rate (R). The analysis revealed a statistically significant and strong negative correlation (r = −0.847, p < 0.0001, R² = 0.717; see Fig 4). This indicates that within the specific context of this study, streets with higher composite environmental scores tended to be associated with lower VSC rates. This observed pattern suggests that certain configurations of environmental factors may coincide with safer street conditions. However, given the observational and geographically focused design of this study, these findings are inherently preliminary; they highlight an association that warrants further investigation in broader and more diverse settings.

The overall score ($\sum {\text{S}}_{\text{i}}$) was derived by first calculating a composite impact index (${\text{S}}_{\text{i}}$) for each design factor, which integrates its relevance weight (${\text{G}}_{\text{i}}$) from the Fuzzy Delphi Method with its site-specific performance score (${\text{a}}_{\text{i}}$). The summation of these ${\text{S}}_{\text{i}}$ values represents the collective influence of multiple environmental attributes. While the identified negative correlation is strong, it should not be interpreted as a purely linear or deterministic relationship. Rather, it reflects a consistent trend wherein streets with higher aggregated design scores generally exhibit lower crime rates, suggesting that the confluence of positively rated environmental factors collectively contributes to a reduction in VSC occurrence. This approach also underscores that different street design factors exert varying degrees of influence on crime patterns, reinforcing the need for a multi-faceted assessment of street environments.

ANOVA was employed to further dissect the differences in the average monthly crime counts across the fifteen streets. The results confirmed statistically significant differences (Welch’s F (14, 295.50) = 45.76, p < 0.001), demonstrating that the distribution of street-level crime is not random but closely linked to specific environmental factors, even within a broadly consistent socio-economic district. Post hoc comparisons using the Games-Howell test delineated three statistically distinct street clusters: a high-crime cluster (Streets A, B, C, O), a medium-crime cluster (Streets D, F, G, I, J, K, L, M, N), and a low-crime cluster (Streets E, H). Notably, no significant differences were found within the high-crime cluster (e.g., Street A vs. B vs. C, p > 0.05), whereas both Streets E and H from the low-crime cluster differed significantly from nearly all streets in the high-crime cluster (p < 0.001). Interestingly, an examination of the low-crime group (e.g., Street E) reveals that high-quality maintenance of shrubs and street trees can sometimes compensate for modest lighting or surveillance. This suggests that well-kept vegetation enhances the perception of care and supports natural surveillance, thereby suppressing criminal opportunities—a finding consistent with Ceccato’s research [28]. Conversely, Street D, a member of the medium-crime cluster, exhibited better lighting and surveillance coverage than Street E but still had a higher crime rate. This underscores the complex, non-substitutable interplay among environmental factors, where the overall configuration, rather than any single element in isolation, determines the crime outcome.

Among the individual factors, “street lights”, “street surveillance”, and “nightclubs/bars” were identified by the FDM as having the highest correlation with VSC rates (${\text{G}}_{\text{i}}\;>\;\text{6}.\text{00}$). This identifies street lighting as a critical factor strongly correlated with VSC rates in this study area, highlighting it as a priority for further investigation and potential intervention in high-crime areas. Similarly, street surveillance strengthens the monitoring of public spaces, deterring potential offenders. The strong link between bars/nightclubs and VSC establishes them as primary concerns for prevention efforts. These deductions align with the findings of Xu, Long, and Hewitt et al. [18,33,58]. Consequently, environments typically associated with VSC in this study include poorly lit areas, side streets with limited monitoring, and establishments selling alcohol, a profile that resonates with Ceccato’s observations [28]. On the other hand, factors such as “motor vehicle/non-motor vehicle parking” and “stores” did not demonstrate a strong or clear-cut association with VSC in this context, indicating that their role is more ambiguous and likely context-dependent.

The ambiguity of factors like “bus stops” and “stores” warrants note. While daytime foot traffic can increase natural surveillance and potentially deter certain crimes, their presence during periods of low activity (e.g., at night) can elevate risks for violent crimes. Similarly, “potholes” do not directly cause violent crime but may signal broader neglect, indirectly increasing crime likelihood through the Broken Windows Theory [21]. This finding aligns with the understanding that crime patterns are highly context-dependent, with certain environments becoming risk hotspots at specific times.

**Fig 5:**
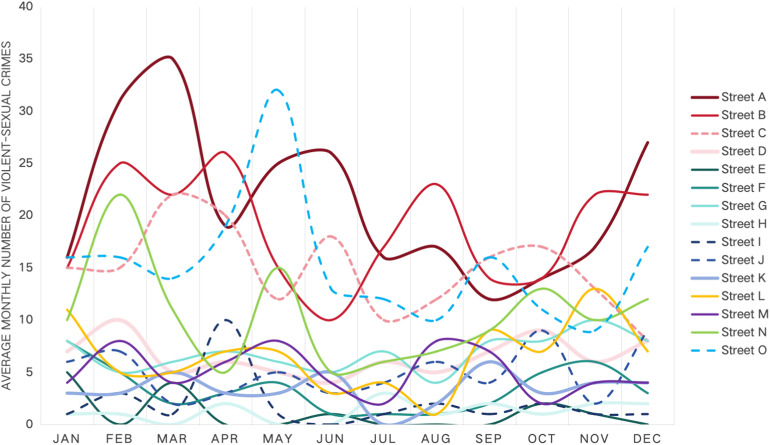
Average monthly number of VSC by street.

Beyond static design, the 53-month data reveals distinct temporal patterns in VSC occurrence (see Fig 5). Crime counts exhibited clear seasonal fluctuations, with peaks frequently occurring in February, May, and November, and troughs more common in January, April, and October. Peaks in February and May may be linked to increased outdoor activity as winter ends or when spring vegetation creates new concealment opportunities. Notably, crime levels remained elevated during the mid-year months (July-August), potentially linked to heat-aggression effects and increased nighttime activity during summer holidays. The November spike may be related to rapidly diminishing daylight hours, providing more cover of darkness, while temperatures remain permissive for outdoor activity. In contrast, the January trough likely reflects post-holiday patterns with reduced outdoor activity, while the April and October lows correspond to periods of mild, stable weather and good environmental visibility, optimizing natural surveillance conditions. These findings emphasize that street safety is a dynamic intersection of physical design, seasonal climate, and human behavioral shifts. Temperature, daylight, and vegetation phenology interact with the physical street environment to shape a calendar of crime risk.

## Conclusions

Street space serves as a critical nexus for urban life, functioning not only as a transit corridor but also as a primary venue for social interaction. The emphasis on safety in the design of urban street space is becoming increasingly evident. The development of a safe street environment significantly enhances residential satisfaction and broader social development. However, numerous crimes still occur on urban streets, and streets with a high crime rate can increase the fear of crime among surrounding residents. Environmental factors in urban street spaces have a crucial impact on the occurrence of crime, and studies have demonstrated that single or multiple environmental factors in street spaces are related to the crime rate. However, few studies have addressed the correlation between overall integrated street design and Violent-Sexual Crimes (VSC).

This research addresses this gap by developing and preliminarily applying a multi-factor evaluative framework to assess street environmental attributes associated with VSC rates. This study focuses on the overall evaluation of street environmental factors. The FDM was used to rank the importance of environmental factors that influence the crime rate of streets. The randomly selected streets were scored using the GSV and Semantic Segmentation to assess the feasibility of the street scoring criteria. ANOVA was used to analyze between-group and within-group differences, and the reasons for the significant differences were discussed. According to the FDM, “street surveillance”, “street lights”, and “nightclubs/bars” are the factors most strongly correlated with VSC in the studied streets. “Bus stops”, “shrub”, and “street trees” are also factors that cannot be ignored. This can be linked to the Defensible Space Theory and leads to the conclusion that VSCs are often committed close to vegetation (poorly maintained plants, street trees); in poorly monitored areas (lack of surveillance or “street eyes”); in places where alcohol is sold; in places with low visibility (low traffic or dimly lit areas); and in places where it is easy to escape the crime scene (near bus stops or surrounded by motor vehicles) [59]. These results may assist urban planners and policymakers in prioritizing the management of key environmental factors in similar contexts and serve as a preliminary tool for crime prevention consideration.

In summary, this study proposes a potential street design framework, and the proposed street design scoring criteria were applied in a specific setting. Within this study, streets with higher scores were associated with lower VSC occurrence. The crime rates of the selected streets provided preliminary evidence for the practicality of this design criteria. In addition, the FDM adopted in this study can be applied to develop other similar design criteria.

Despite the fact that this research is based in London and that different cities or regions have their own distinct characteristics, the methodological framework and some context-specific findings may offer insights for other regions. For example, this paper suggests that the three factors most associated with VSC - “surveillance”, “lights,” and “nightclubs” – could be considered as potential focal points in planning and design, subject to local validation. Some ambiguous factors, such as “bus stops”, “stores” and “potholes”, should be evaluated and adapted according to the location and culture of the area. This highlights the need for contextual adaptation of any design guidelines.

This study is an exploratory analysis of street-level environmental factors within a specific context. The primary limitation stems from the sampling strategy and sample size, which may affect the statistical power to detect subtle relationships between environmental factors and VSC rates. While the sampling aimed for diversity within this bounded area, it does not represent the broader variability of London or other cities, and findings should be interpreted as preliminary. This limits the direct applicability and generalizability of the specific scoring criteria and environmental factor weights to cities with different cultural contexts and urban layouts. Additionally, as noted in the Methods section, our analysis relied on raw crime counts rather than rates normalized by street length or pedestrian activity, which limits the precision of cross-street comparisons. Future research should incorporate normalized metrics (e.g., crimes per kilometer or per pedestrian-hour) to facilitate more robust and comparable analyses. Furthermore, as an observational study, it cannot control for all potential confounding factors (e.g., transient population flows). Therefore, claims regarding robustness and universality are intentionally tempered. Furthermore, although the expert panel involved in the FDM possessed expertise in environmental design, urban planning, and related areas such as sociology and Crime Prevention Through Environmental Design (CPTED), further exploration can involve more experts with diverse backgrounds. Future research would benefit from integrating broader interdisciplinary perspectives to deepen the understanding of VSC-specific risk factors. The main contribution of this work lies in proposing and providing preliminary evidence for a structured, multi-factor assessment framework within a defined setting. The findings highlight associations in this specific context and establish a foundation for future hypothesis testing in broader, more diverse environments. Subsequent studies should prioritize applying this framework across multiple cities to examine its broader utility.

## Supporting information

S1 Questionnaire Fuzzy Delphi Method (FDM) questionnaire. (DOC)

S1 Table Results of Semantic Segmentation. (CSV)

S2 Table Street environment factor scores for the fifteen studied streets. (XLSX)

S3 Table Violent-Sexual Crimes quantities on streets. (XLSX)

S4 Table Post-hoc analysis (Games-Howell test) results. (XLSX)
