# Supplementary material for: How can we reduce the rate of Violent-Sexual Crimes? An attempt at street design
Source: PLoS One. 2026 Jan 21;21(1):e0341372. doi: 10.1371/journal.pone.0341372 (PMC12822935; doi:10.1371/journal.pone.0341372)
Supplement: S1 Questionnaire — (DOC) [file pone.0341372.s001.doc]

**Supplementary: FDM Questionnaire**

The purpose of this questionnaire is to evaluate the extent to which various environmental factors are associated with violent and sexual crime rates in street spaces. This assessment aims to determine whether a specific factor influences changes in crime rates while other street environmental factors remain constant.

A rating scale is used for scoring, as follows:

0 – Not related at all

3 – Slightly related

5 – Moderately related

8 – Significantly related

10 – Necessarily related

Scores range from 0 to 10, with higher scores indicating a stronger perceived relationship. For each item, please provide:

**Acceptable Minimum:** the lowest score you consider appropriate

**Your Expert Value:** the score that best reflects your expert judgment

**Acceptable Maximum:** the highest score you consider appropriate

1. **Shrubs:** enhance shrub maintenance.

Acceptable Minimum:

Your expert value:

Acceptable Maximum:

1. **Street trees:** enhance street tree maintenance.

Acceptable Minimum:

Your expert value:

Acceptable Maximum:

1. **Lighting:** increase the number of street lights.

Acceptable Minimum:

Your expert value:

Acceptable Maximum:

1. **Surveillance:** reduce the number of motorized or non-motorized vehicles parking spaces.

Acceptable Minimum:

Your expert value:

Acceptable Maximum:

1. **Surveillance:** increase the amount of street surveillance.

Acceptable Minimum:

Your expert value:

Acceptable Maximum:

1. **Maintenance:** reduce potholes.

Acceptable Minimum:

Your expert value:

Acceptable Maximum:

1. **Maintenance:** reduce litter on the street.

Acceptable Minimum:

Your expert value:

Acceptable Maximum:

1. **Building Façade:** increase personalized house or yard decorations.

Acceptable Minimum:

Your expert value:

Acceptable Maximum:

1. **Building Façade:** reduce exterior stains or peeling concrete.

Acceptable Minimum:

Your expert value:

Acceptable Maximum:

1. **Sign:** reduce the number of bus stops.

Acceptable Minimum:

Your expert value:

Acceptable Maximum:

1. **Activity:** reduce the number of street stores.

Acceptable Minimum:

Your expert value:

Acceptable Maximum:

1. **Activity:** reduce the number of schools.

Acceptable Minimum:

Your expert value:

Acceptable Maximum:

1. **Activity:** reduce the number of nightclubs or bars.

Acceptable Minimum:

Your expert value:

Acceptable Maximum:

**What other factors do you believe influence street crime?**

1.___________________________________

2.___________________________________

3.___________________________________

4.___________________________________

Thank you very much! **Please provide your information below.**

Name (optional): ______________________

Major (*required): _____________________________

Academic qualification (*required): _____________________

Thank you for your participation. This survey is intended for academic use only. Your feedback is highly valuable and will help guide and refine this research. We sincerely appreciate your time and expert input.
